# Anthocyanin Protects Cardiac Function and Cardiac Fibroblasts From High-Glucose Induced Inflammation and Myocardial Fibrosis by Inhibiting IL-17

**DOI:** 10.3389/fphar.2020.593633

**Published:** 2021-02-02

**Authors:** Er Yue, Yahan Yu, Xinyao Wang, Bing Liu, Yunlong Bai, Baofeng Yang

**Affiliations:** ^1^ Department of Pharmacology (State-Province Key Laboratories of Biomedicine- Pharmaceutics of China, Key Laboratory of Cardiovascular Medicine Research, Ministry of Education), College of Pharmacy, Harbin Medical University, Harbin, China; ^2^ Chronic Disease Research Institute, Translational Medicine Research and Cooperation Center of Northern China, Heilongjiang Academy of Medical Sciences, Harbin, China

**Keywords:** anthocyanin, diabetic cardiomyopathy, IL-17, inflammation, myocardial fibrosis

## Abstract

Diabetic cardiomyopathy (DCM) is one of the major causes of death in diabetic patients. Its pathogenesis involves inflammation and fibrosis that damages the heart tissue and impairs cardiac function. Interleukin (IL)-17, a pro-inflammatory cytokine that plays an important role in a variety of chronic inflammatory processes can serve as an attractive therapeutic target. Anthocyanin, a water-soluble natural pigment, possesses impressive anti-inflammatory activity. However, its role in DCM is unclear. Hence, we investigated the protective effect of anthocyanin on the cardiovascular complications of diabetes using a mouse type 1 diabetes mellitus model induced by streptozotocin. Cardiac function and structural alterations in diabetic mice were tested by echocardiography, hematoxylin and eosin staining, and Masson trichrome staining. Immunohistochemistry was performed to evaluate the distribution and deposition of IL-17 and collagen I and III from the left ventricular tissues of diabetic mice. Cell viability was measured using the methyl thiazolyl tetrazolium assay. Protein levels of IL-17, tumor necrosis factor α, IL-1β, and IL-6 were determined using enzyme-linked immunosorbent assay. IL-17 and collagen I and III were detected by western blotting and immunofluorescence, and their mRNA levels were quantified using quantitative reverse transcription PCR. We observed that anthocyanin lowered blood glucose, improved cardiac function, and alleviated inflammation and fibrosis in the heart tissue of diabetic mice. Meanwhile, anthocyanin reduced the expression of IL-17 in high-glucose-treated cardiac fibroblasts and exhibited an anti-inflammatory effect. Deposition of collagen I and III was also decreased by anthocyanin, suggesting that anthocyanin contributes to alleviating myocardial fibrosis. In summary, anthocyanin could protect cardiac function and inhibit IL-17-related inflammation and fibrosis, which indicates its therapeutic potential in the treatment of diabetes mellitus-related complications.

## Introduction

Diabetic cardiomyopathy (DCM) is a serious cardiovascular complication of both type 1 and type 2 diabetes mellitus (DM) that accounts for a significant proportion of deaths in diabetic patients worldwide (Abdelsamia et al., 2019; Sadek et al., 2020). The pathophysiological course of DCM presents with cardiac dysfunction independent of coronary artery disease, hypertension, and valvular heart disease (Dillmann, 2019). Among two types of DM, type 1 DM shows more obvious myocardial inflammation and fibrosis associated with oxidative stress and apoptotic processes, which leads to systolic dysfunction and concentric left ventricular hypertrophy compared with type 2 DM (Radovits et al., 2015; Palomer et al., 2018). Among the numerous pathogenic factors, inflammation induced by high blood glucose is known to play a critical role in the pathogenesis of DCM. Therefore, suppression of inflammation may improve cardiac function and reduce the mortality rate in diabetic patients.

Interleukin-17A (IL-17A), originally known as the cytotoxic T lymphocyte-associated antigen 8 (CTLA-8), is a pro-inflammatory cytokine (Rouvier et al., 1993), and has emerged as a new therapeutic target in chronic inflammatory diseases, such as psoriasis (Karbach et al., 2014) and vitiligo (Zhou et al., 2018). It has been reported that IL-17 is involved in the development of some cardiovascular diseases (Liao et al., 2012), including atherosclerotic cardiovascular disease (Gong et al., 2015) and myocardial injury (Zhou et al., 2014). DCM manifests as myocardial fibrosis and is often aggravated by inflammation with increased macrophage and leukocyte infiltration as well as the upregulation of inflammatory cytokines (Rajesh et al., 2012). Recently, it has been shown that IL-17 contributes to the development of myocardial fibrosis (Li et al., 2019b). A number of studies have also revealed the relationship between microRNAs (miRNAs) and IL-17; miR-125a-3p decreases the level of IL-17 and suppresses renal fibrosis (Zhang et al., 2019), while miR-340 alleviates psoriasis in mice through direct targeting of IL-17 (Bian et al., 2018). Meanwhile, we showed that miR-214-3p participates in the regulation of DCM in diabetic mice in our previous study (Yang et al., 2018; Yang et al., 2019). Therefore, we speculated that miR-214-3p also participates in the regulation of IL-17 in DCM.

Anthocyanin is a natural active ingredient found in a variety of plants and consists of aglycons attached to glycosyl groups (Blesso, 2019). Anthocyanin is well known for its strong antioxidant property. The anti-tumor activity of anthocyanin has also been widely recognized (Peiffer et al., 2016). Moreover, this compound also possesses appreciable anti-inflammatory (Daveri et al., 2018), anti-hyperglycemic (Roopchand et al., 2013), and anti-fibrotic properties (Morrison et al., 2015). Our previous study revealed its cardioprotective role by demonstrating that anthocyanin could attenuate myocardial ischemia-induced injury via the inhibition of the ROS-JNK-Bcl-2 pathway (Syeda et al., 2019). However, its role in DCM is still unclear. Therefore, in this study, we sought to investigate whether anthocyanin could improve cardiac function in diabetic mice considering its anti-inflammatory and anti-fibrotic properties, and whether the inhibition of IL-17 might possibly contribute to the beneficial action of this compound.

## Materials and Methods

### Anthocyanin

Anthocyanin was obtained from Lingoberry Boreal Biotech Co., Ltd. (DaXingAnLing, China). High performance liquid chromatography was performed to prove that active ingredient of anthocyanin is cyanidin-3-O glucoside, as described in our previous study (Yue et al., 2019).

### Establishment of the Diabetic Mouse Model

Male C57BL/6 mice weighing 20–24 g were purchased from Liaoning Changsheng Biotechnology Co., Ltd. (Liaoning, China). The mice were randomly divided into the following three groups: control group (Control), diabetes mellitus group (DM), and DM treated with anthocyanin group (DM + AC). Mice were administered once with an intraperitoneal injection of 150 mg/kg streptozotocin (STZ, Sigma, MO, United States) dissolved in citrate buffer (pH = 4.6). Mice with blood glucose >16.7 mmol/L were considered diabetic. Mice in the DM + AC group were administered 250 mg/kg of anthocyanin every day by an intragastric gavage (Chen et al., 2016). All mice were maintained for 12 weeks (n = 5 for each group).

### Primary Cell Culture and Transfections

Cardiac fibroblasts isolated from C57BL/6 neonatal mice were cultured in Dulbecco’s Modified Eagle’s Medium (DMEM) (HyClone, Logan, UT, United States) supplemented with 10% fetal bovine serum (FBS) (Gibco, Thermo Fisher Scientific, MA, United States) at 37°C and 5% CO_2_, and treated with 5.5 mM glucose (NG), 25 mM glucose (HG), or 25 mM glucose with 250 μg/ml anthocyanin (HG + AC) for 48 h. X-treme GENE siRNA transfection reagent (Roche, Germany) was used for transfection with anti-miR-214-3p oligonucleotides (AMO-214-3p) with corresponding negative controls (AMO-NC) designed and synthesized by RIOBIO (Guangzhou, China). This procedure was performed in accordance with the manufacturer’s instructions. The sequence of the miR-214-3p inhibitor was 5′-mAmCmUmGmCmCmUmGmUmCmUmGmUmGmCmCmUmGmCmUmGmUmGmU-3′.

### MTT Assay

The viability of cardiac fibroblasts was determined using the MTT assay (Promega, WI, United States). Cardiac fibroblasts in 200 μL cultured medium containing 10% FBS were incubated overnight at 37°C and 5% CO_2_. The medium was replaced with differing concentrations of anthocyanin (0, 62.5, 125, 250, and 500 μg/ml). After treatment over different time points (24 and 48 h), each well was replaced with 100 μL of medium containing 10 μL of MTT solution followed by incubation in total darkness. Four hours later, the absorbance was measured at 450 nm using a microplate reader.

### Echocardiography

Twelve weeks later, mice were subjected to avertin-induced anesthesia after depilation. The following structural variables were evaluated by M-Mode from a Vevo1100 high-resolution imaging system (VisualSonics, Toronto, ON, Canada): interventricular septal thickness in systole (IVSs) and diastole (IVSd), left ventricular internal dimension in diastole (LVIDd), left ventricular internal dimension in systole (LVIDs), and LV posterior wall thickness in systole (LVPWs) and diastole (LVPWd) and LV mass. LV function was assessed by the following parameters, including left ventricular fractional shortening (LVFS) and ejection fraction (LVEF).

### Total RNA Isolation and Quantitative Reverse Transcription PCR

RNA samples from mice myocardial tissues and neonatal mice cardiac fibroblasts were isolated using TRIzol reagent (Invitrogen, CA, United States). RNA was reverse transcribed to cDNA using the Toyobo reverse transcription reagent kit (Toyobo, Osaka, Japan). Subsequently, qRT-PCR was performed using the Toyobo SYBR qPCR mix reagent (Toyobo, Osaka, Japan) and ABI 7500 fast Real-Time PCR system (Applied Biosystems, CA, United States). The sequences of the primers used are listed in Table 1. The relative quantification of the gene expression levels was carried out using the 2^−ΔΔCT^ method.

**TABLE 1:** PCR primer sequence.

Primer		RNA sequence
GAPDH	Forward	5′-ATC​ACT​GCC​ACC​CAG​AAG​AC-3′
	Reverse	5′-TTT​CTA​GAC​GGC​AGG​TCA​GG-3′
IL-17	Forward	5′-ACC​GCA​ATG​AAG​ACC​CTG​AT-3′
	Reverse	5′-CAG​GAT​CTC​TTG​CTG​GAT​GAG​A-3′
Collagen I	Forward	5′-GCC​CTT​CTG​GTC​CTA​TTG​G-3′
	Reverse	5′-CTA​CCA​GTG​TTG​CCA​GTG​TC-3′
Collagen III	Forward	5′-CCC​CTG​GTT​CTT​CTG​GAC​AT-3′
	Reverse	5′-CCT​GAC​TCT​CCA​TCC​TTT​CCA-3′

### Protein Extraction and Western Blotting

Protein samples from mice myocardial tissues and neonatal mice cardiac fibroblasts were extracted using RIPA buffer and separated on a 12% gel by sodium dodecyl sulfate-polyacrylamide gel electrophoresis. The proteins were then transferred onto polyvinylidene difluoride membranes (Millipore, MA, United States) and blocked with 5% non-fat dry milk for 2 h at room temperature. Membranes were incubated with primary antibodies against IL-17 (Santa Cruz Biotech, CA, United States), collagen I (Abcam, Cambridge, United Kingdom), collagen III (Abcam, Cambridge, United Kingdom), and GAPDH (ZSGB-BIO, Beijing, China) (1:1,000) at 4°C overnight followed by incubation with goat anti-mouse immunoglobulin G (IgG) (ZSGB-BIO, Beijing, China) or anti-rabbit IgG (ZSGB-BIO, Beijing, China) at room temperature for 1 h. GAPDH was used as the internal control.

### Hematoxylin and Eosin Staining and Masson Trichrome Staining

Left ventricular tissues from diabetic mice were fixed in 4% paraformaldehyde for dehydration, and paraffin sections were prepared. Staining was carried out according to the manufacturer’s instructions provided in the HE and Masson staining kit (Solarbio, Beijing, China). Morphological and collagen changes were analyzed under a fluorescence microscope (Nikon 80i, Tomu Prefecture, Otawara, Japan).

### Immunohistochemistry

Left ventricular samples were fixed in 4% paraformaldehyde. Paraffin sections were prepared and stained with IL-17, collagen I, and collagen III primary antibodies (1:200) overnight at 4°C, followed by incubation with secondary antibodies. Subsequently, the sections were stained with diaminobenzidine, and the images were captured using a fluorescence microscope.

### Enzyme-Linked Immunosorbent Assay

The levels of IL-17, TNF-α, IL-1β, and IL-6 were measured in the samples prepared from the supernatant of cultured cardiac fibroblasts and serum of mice as per the manufacturer’s instructions provided in the ELISA kit (Boster Bio Tech, Wuhan, China).

### Immunofluorescence

Cardiac fibroblasts were fixed with 4% paraformaldehyde for 15 min, followed by the addition of 0.1% Triton X-100 for 5 min. The reaction was blocked using 5% BSA at room temperature for 1 h. The samples were incubated with the primary antibodies of IL-17, collagen I, and collagen III (1:200) overnight at 4°C. Subsequently, the samples were incubated with secondary antibody at 37°C for 1 h, followed by incubation with DAPI for 15 min. Images were captured using a fluorescence microscope.

### Statistical Analysis

Graphs were plotted using the GraphPad Prism 5.0 software. Data are expressed as mean ± standard deviation (mean ± SD). Statistical comparisons among multiple groups were performed using the one-way analysis of variance (ANOVA) followed by Tukey’s post hoc test. A value of *p* < 0.05 was considered statistically significant.

## Results

### Anthocyanin Decreases Blood Glucose and Improves Cardiac Function in Diabetic Mice

**FIGURE 1:**
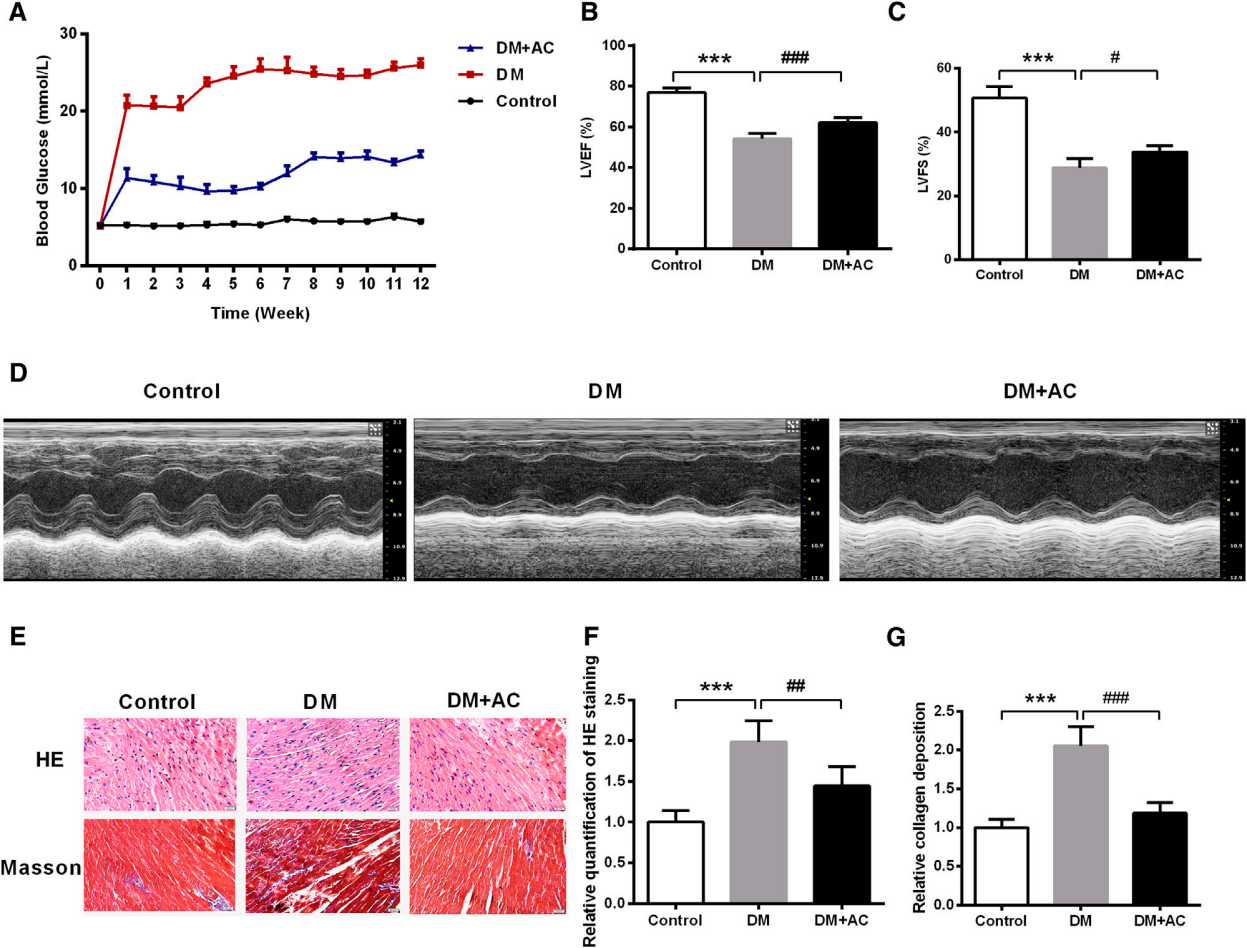
Anthocyanin reduces blood glucose and improves cardiac function in diabetic mice. **(A)** Blood glucose from 0 to 12 weeks. **(B)** Left ventricle ejection fraction (LVEF) and **(C)** Left ventricular fractional shortening (LVFS) are shown. **(D)** M-mode echocardiograms of left ventricle. **(E)** Hematoxylin and eosin (HE) and Masson’s trichrome staining. **(F)** Relative quantification of HE staining. **(G)** Relative collagen deposition. Scale bar, 50 μm. ***p < 0.001 vs. Control, ##*p* < 0.01 vs. diabetes mellitus (DM); ###*p* < 0.001 vs. DM; n = 5.

We established an STZ-induced type 1 diabetes mouse model. The blood glucose level in the DM + AC group was found to be decreased compared to that in the DM group (Figure 1A). Subsequently, the echocardiographic measurement of cardiac function was carried out. As shown in Figures 1B–D and Supplementary Figures S2A–G, there were no significant differences in the values of IVDd, LVIDd, and LVPWd among the three groups. However, LVIDs was increased, while IVSs, LVPWs, LVEF, LVFS and LV mass were decreased in the DM group compared to that in the control group. After treatment with anthocyanin, LVPWs, LVEF, LVFS, and LV mass were found to be restored in the DM + AC group. In summary, anthocyanin significantly improved cardiac function in diabetic mice.

HE and Masson trichrome staining demonstrated that the myocardial mass improved, while collagen deposition decreased after administration of anthocyanin in the DM group (Figures 1E–G).

### Anthocyanin Attenuates Inflammation and Myocardial Fibrosis in Diabetic Mice

**FIGURE 2:**
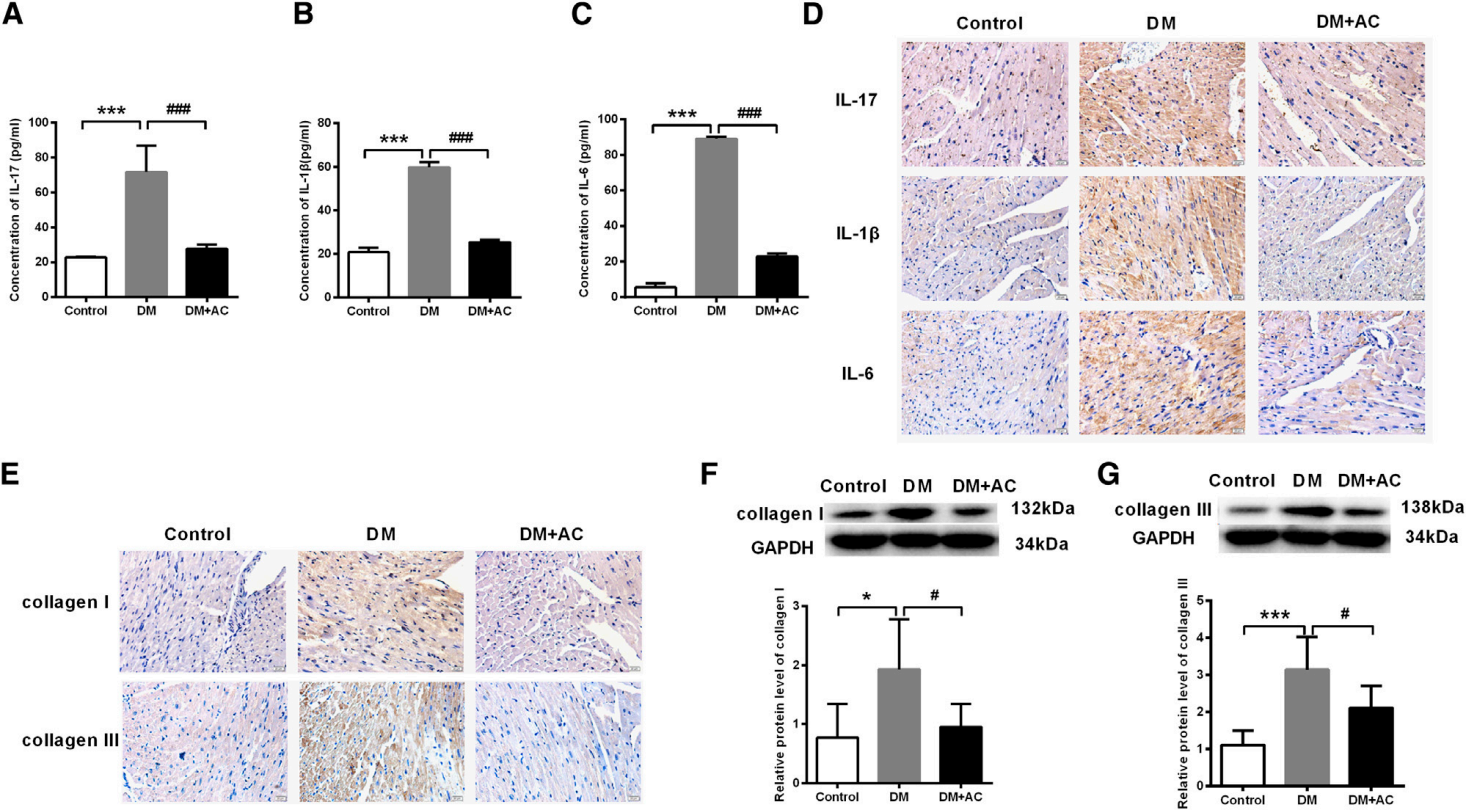
Anthocyanin attenuates inflammation and myocardial fibrosis in diabetic mice. **(A)** Concentration of interleukin (IL)-17 detected by enzyme-linked immunosorbent assay (ELISA). **(B)** Concentration of IL-1β detected by ELISA. **(C)** Concentration of IL-6 detected by ELISA. **(D)** Immunohistochemistry analysis of IL-17 (first lane), IL-1β (second lane), and IL-6 (third lane). Scale bar: 20 μm. **(E)** Immunohistochemistry analysis of collagen I (first line) and III (second line). Scale bar: 20 μm. **(F)** Relative protein level of collagen I. **(G)** Relative protein level of collagen III. GAPDH served as an internal control. *p < 0.05 vs. Control, ***p < 0.001 vs. Control, #*p* < 0.05 vs. diabetes mellitus (DM), ###*p* < 0.001 vs. DM; n = 5.

Results from ELISA of the diabetic mice serum showed that the elevation in the levels of IL-17, IL-1β, and IL-6 in the DM group were inhibited by anthocyanin (Figures 2A–C), which was consistent with the immunohistochemical analysis of the cardiac tissues (Figure 2D). Similarly, accumulation of collagen I and III was also inhibited by anthocyanin in the DM + AC group compared to that in the DM group (Figure 2E). Moreover, anthocyanin also reduced the protein levels of collagen I and III in DM mice (Figures 2F,G). These results suggest that anthocyanin exhibits anti-inflammatory and anti-fibrotic effects that improve the cardiac function of diabetic mice.

### Anthocyanin Reduces High Glucose-Induced Upregulation of Interleukin-17 in Cardiac Fibroblasts

**FIGURE 3:**
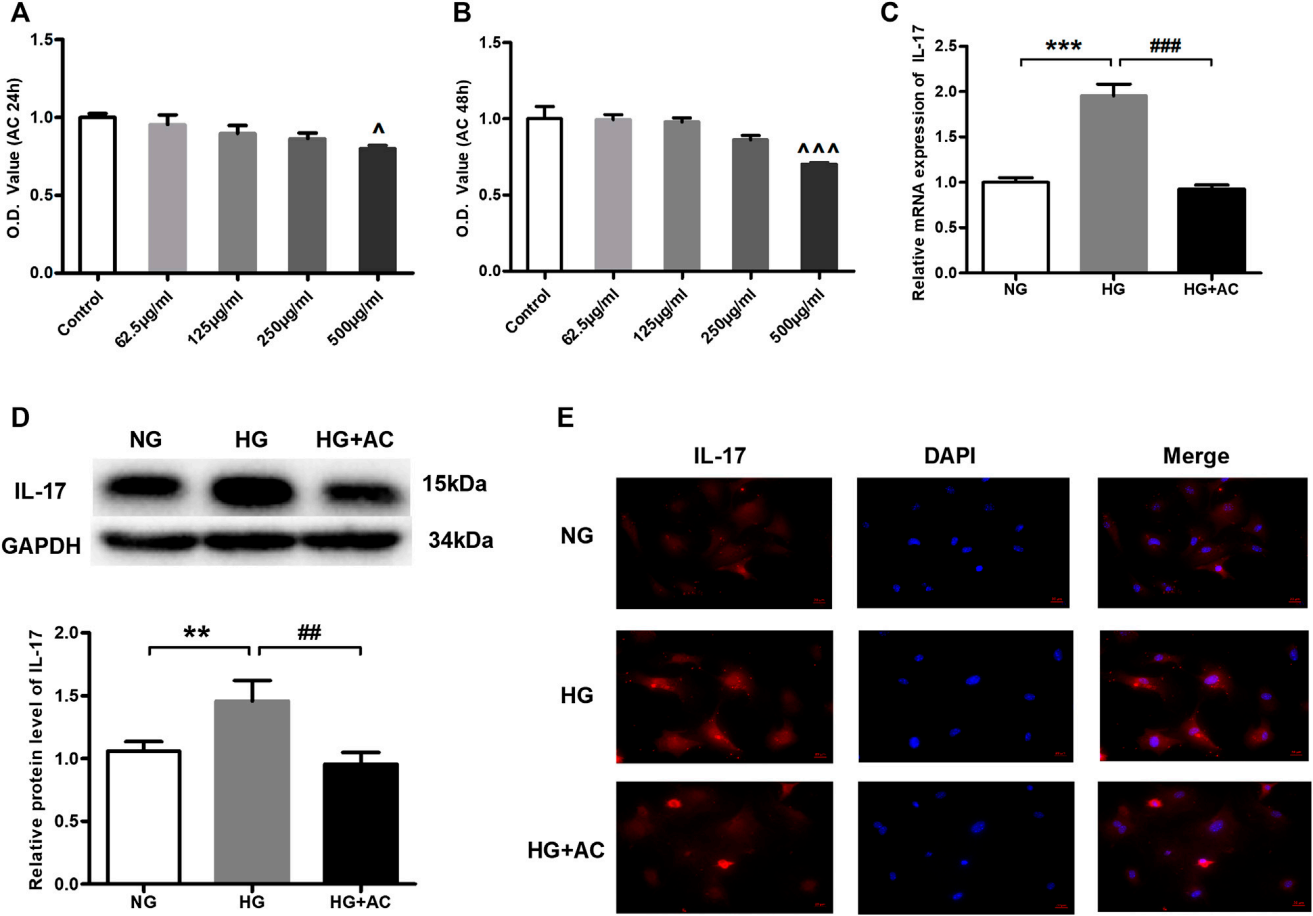
Anthocyanin represses high glucose-induced abnormal upregulation of interleukin (IL)-17 expression in cardiac fibroblasts isolated from neonatal mice. **(A)** Alterations in viability at 24 h. **(B)** Alterations in viability at 48 h. **(C)** Relative expression of IL-17 mRNA detected using quantitative reverse transcription PCR (qRT-PCR). **(D)** Expression of IL-17 protein. GAPDH served as an internal control. **(E)** Immunofluorescence staining of IL-17. Scale bar, 20 μm. ^*p* < 0.05 vs. Control, ^^*p* < 0.01 vs. Control, ^^^*p* < 0.001 vs. Control, ***p < 0.001 vs. NG, ###*p* < 0.001 vs. HG. NG, normal glucose (5.5 mM); HG, high glucose (25 mM); HG + AC, high glucose (25 mM) + anthocyanin (250 μg/ml); n = 5 for A-C, n = 3 for D-F.

To further explore the protective effect of anthocyanin on the cardiac function of diabetic mice, we conducted the following study in HG-induced cardiac fibroblasts. To select a concentration and treatment time-point of anthocyanin that is not cytotoxic to the cardiac fibroblasts, we first performed MTT assays to assess the changes in cell viability in the presence of varying concentrations of anthocyanin (62.5, 125, 250, and 500 μg/ml) at varying time points (24 and 48 h). We found that anthocyanin causes a significant decrease in the cell viability of cardiac fibroblasts at both 24 and 48 h only at a concentration of 500 μg/ml (Figures 3A,B). Therefore, our subsequent experiments were carried out with 250 μg/ml anthocyanin treatment for 24 h. Next, we compared the levels of IL-17 in the HG group, the HG with 250 μg/ml anthocyanin (HG + AC) group, and the normal glucose (NG) group. The results showed that both the mRNA (Figure 3C) and protein levels (Figure 3D) of IL-17 increased in the HG group and were effectively reduced following anthocyanin treatment. The immunofluorescence results of the cardiac fibroblasts are consistent with the above results (Figure 3E). These findings indicate that anthocyanin can reduce the upregulation of IL-17 induced by HG in cardiac fibroblasts.

### Anthocyanin Attenuates Myocardial Fibrosis and the Levels of Inflammatory Cytokines Induced by High Glucose in Cardiac Fibroblasts

**FIGURE 4:**
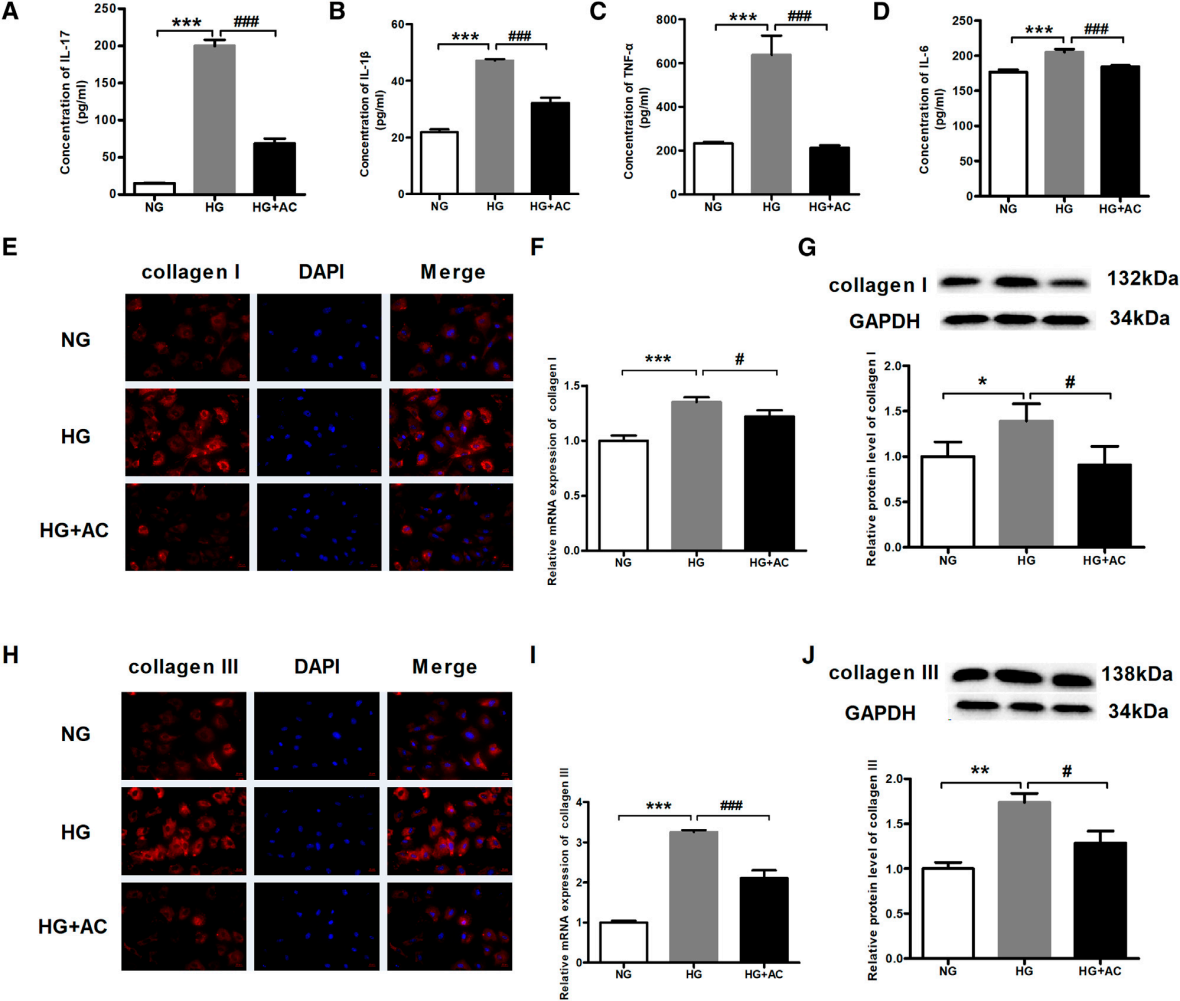
Anthocyanin reduces myocardial fibrosis and the levels of inflammatory cytokines in cardiac fibroblasts from neonatal mice induced by high glucose. **(A)** Concentration of interleukin (IL)-17 detected by enzyme-linked immunosorbent assay (ELISA). **(B)** Concentration of IL-1β detected by ELISA. **(C)** Concentration of TNF-α detected by ELISA. **(D)** Concentration of IL-6 detected by ELISA. **(E)** The immunofluorescence staining of collagen I. Scale bar: 20 μm. **(F)** Relative expression of collagen I mRNA detected using quantitative reverse transcription PCR (qRT-PCR). **(G)** Relative protein level of collagen I. **(H)** The immunofluorescence staining of collagen III. Scale bar: 20 μm. **(I)** Relative expression of collagen III mRNA. **(J)** Relative protein level of collagen III. GAPDH served as an internal control. *p < 0.05 vs. NG, **p < 0.01 vs. NG, ***p < 0.001 vs. NG, #*p* < 0.05 vs. HG, ###*p* < 0.001 vs. HG. NG, normal glucose (5.5 mM); HG, high glucose (25 mM); HG + AC, high glucose (25 mM) + anthocyanin (250 μg/ml); n = 3.

Next, we employed ELISA to investigate the effects of anthocyanin on the protein contents of IL-17, IL-1β, TNF-α, and IL-6 due to their synergistic effect on inflammation (Beringer et al., 2016). We found that the IL-17, IL-1β, TNF-α, and IL-6 levels increased in HG-treated cardiac fibroblasts but decreased upon treatment with anthocyanin (Figures 4A–D). Inflammation and diabetes can promote an increase in collagen formation resulting in anatomic and physiological changes in the myocardium, including myocardial fibrosis, which is one of the most frequently proposed mechanisms to explain cardiac changes in DCM (Regan et al., 1981; Goyal and Mehta, 2013; Zhu et al., 2019). Immunofluorescence was, therefore, performed to analyze the abundance and distribution of collagen I and III. The results showed that the fluorescence in the HG group was stronger than that in the HG + AC group (Figures 4E,H). In parallel, we also used qRT-PCR and western blotting to quantify the mRNA and protein expression of collagen I and III. Consistent with the above results, the expression of collagen I (Figures 4F,G) and III (Figures 4I,J) increased in HG-induced cardiac fibroblasts, which was reversed by anthocyanin.

### Anti-Inflammatory and Anti-myocardial Fibrosis Effects of Anthocyanin are Mediated by miR-214-3p and Interleukin-17

**FIGURE 5:**
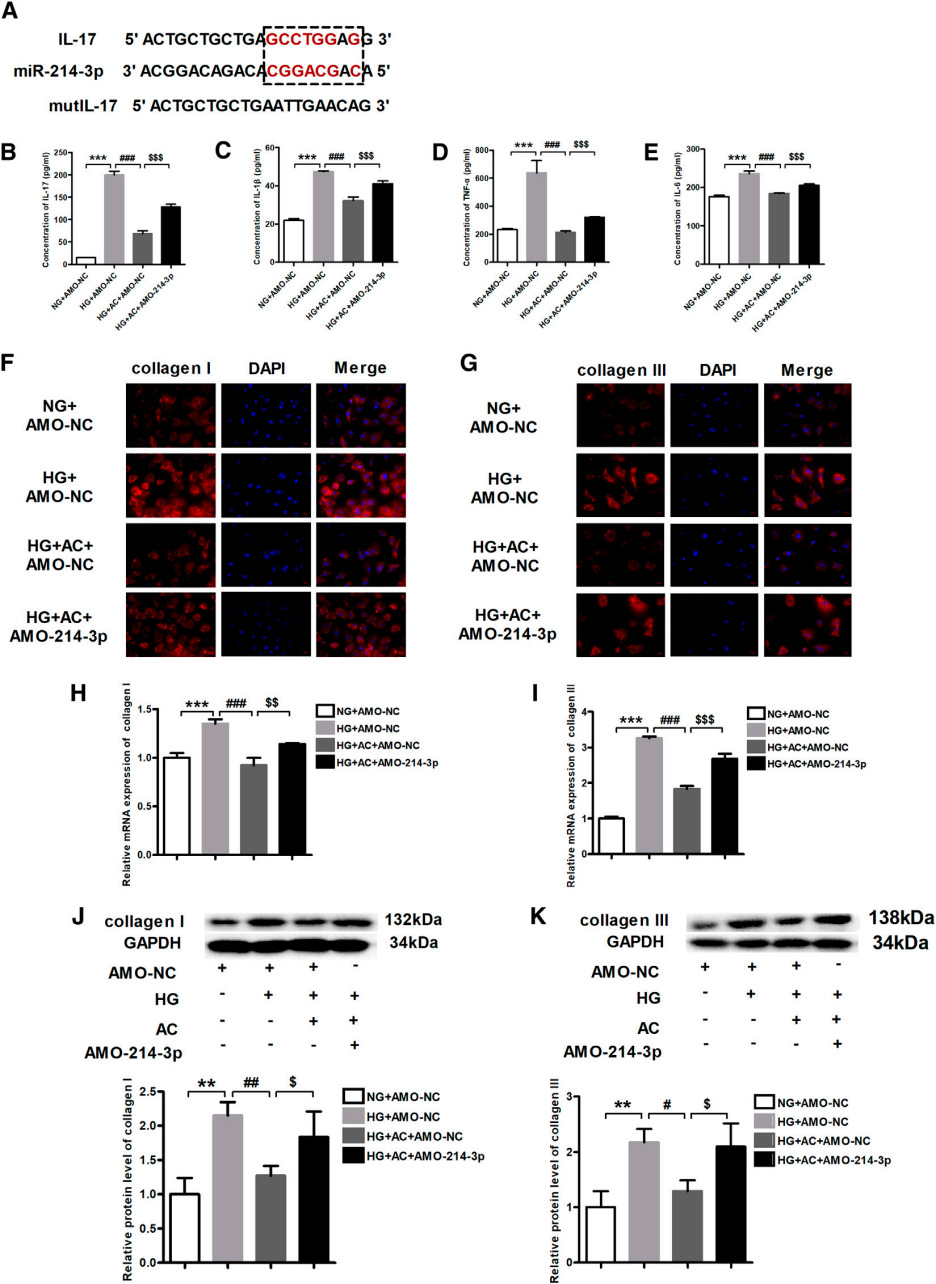
Anthocyanin suppresses inflammation and myocardial fibrosis by inhibiting interleukin (IL)-17 through miR-214-3p. **(A)** Binding targets between miR-214-3p and IL-17. **(B)** Concentration of IL-17 detected by enzyme-linked immunosorbent assay (ELISA). **(C)** Concentration of IL-1β detected by ELISA. **(D)** Concentration of TNF-α detected by ELISA. **(E)** Concentration of IL-6 detected by ELISA. **(F)** Immunofluorescence staining of collagen I. Scale bar: 20 μm. **(G)** Immunofluorescence staining of collagen III. Scale bar: 20 μm. **(H)** Relative expression of collagen I mRNA detected using quantitative reverse transcription PCR (qRT-PCR). **(I)** Relative expression of collagen III mRNA. **(J)** Relative protein level of collagen I. **(K)** Relative protein level of collagen III. GAPDH served as an internal control. **p < 0.01 vs. NG + AMO-NC, ***p < 0.001 vs. NG + AMO-NC, #*p* < 0.05 vs. HG + AMO-NC, ##*p* < 0.01 vs. HG + AMO-NC, ###*p* < 0.001 vs. HG + AMO-NC, $*p* < 0.05 vs. HG + AC + AMO-NC, $$*p* < 0.01 vs. HG + AC + AMO-NC, $$$*p* < 0.001 vs. HG + AC + AMO-NC. NG + AMO-NC, normal glucose (5.5 mM) + AMO-Negative control; HG + AMO-NC, high glucose (25 mM) + AMO-NC; HG + AC + AMO-NC, 25 mM glucose and 250 μg/ml anthocyanin + AMO-NC; n = 3.

As per the findings of a previous study (Qi et al., 2020), miR-214-3p was shown to target IL-17 (Figure 5A). Therefore, we proposed that the observed repression in the expression of IL-17 by anthocyanin might be mediated by miR-214-3p. To this end, we transfected the miR-214-3p antisense inhibitor, AMO-214-3p, into cardiac fibroblasts pretreated with anthocyanin under HG conditions. We subsequently used ELISA to determine the levels of IL-17, IL-1β, TNF-α, and IL-6 in cells of various groups (NG + AMO-NC, HG + AMO-NC, HG + AC + AMO-NC, and HG + AC + AMO-214-3p). The results clearly demonstrated that AMO-214-3p effectively reversed the ability of HG + AC to reduce the HG-induced elevation of the levels of IL-17, IL-1β, TNF-α, and IL-6 (Figures 5B–E).

On the other hand, the immunofluorescence results revealed that the HG-induced production of collagen I and III was attenuated by anthocyanin; however, AMO-214-3p abolished this beneficial effect of anthocyanin (Figures 5F,G). Consistently, while the HG-induced increase in the mRNA and protein levels of collagen I and III were reversed by anthocyanin, the addition of AMO-214-3p eliminated the action of anthocyanin (Figures 5H–K). Taken together, these findings indicate that anthocyanin can exhibit anti-inflammatory and anti-fibrotic effects through IL-17, which seems to play a role through the potential connection between miR-214-3p and IL-17.

## Discussion

DCM originally referred to a pathophysiological course that is caused by myocardial fibrosis, hypertrophy, or microvascular diseases during DM. Subsequently, the contractile dysfunction of cardiomyocytes was added to the definition of DCM to reflect a more precise understanding of the condition (Penpargkul et al., 1981). Currently, there is no unified clinical diagnostic standard for DCM. It is generally believed that patients can be diagnosed with DM and impaired cardiac function, excluding heart failure caused by other heart diseases. Anthocyanin was initially known for its powerful antioxidant effects, but accumulating evidence has proven the positive effects of anthocyanin in cardiovascular diseases. Therefore, we investigated the cardioprotective role of anthocyanin in diabetic mice. We established an STZ-induced type I diabetes mouse model; the diabetic mice fed for three months did not show any chronic toxicity with anthocyanin treatment until sacrifice. The half-life of STZ is 19 minutes (Jensen et al., 1977). Under normal conditions, the drug would be completely metabolized out of the body after five half-lives. In order to eliminate the damage and toxicity of STZ to the heart in this experiment, the heart function test on the mice was performed one week after STZ was administered (Supplementary Figures S1A–C). It was found that after STZ injections, STZ went through a week of metabolic clearance in the body. Although the blood glucose was significantly increased, the heart function was not reduced, and the myocardial tissue had not been damaged (Supplementary Figure S1D), indicating that the STZ treatment did not cause any obvious damage to the heart during the process in the body. The typical feature of DCM is induced after 12 weeks of stable hyperglycemia, indicating that high glucose was the main cause of DCM in this study.

The cardiac fibroblasts from the neonatal mice cultured *in vitro* were treated with high glucose to observe the protective effect of anthocyanin. It was found that the expression of inflammatory factors, collagen I and III, in the cardiac fibroblasts cultured with high glucose were elevated, thereby suggesting that the high glucose environment is one of the main pathogenic factors for inflammatory reaction and fibrosis of cardiac fibroblasts. Anthocyanin inhibited the inflammation and collagen production of cardiac fibroblasts under the condition of continuous high glucose, which indicated that anthocyanin exhibited direct anti-inflammatory and anti-fibrotic ability on cardiac fibroblasts. We found that anthocyanin could reduce blood glucose at the same time, and was accompanied by improving cardiac inflammation and fibrosis in DCM mice. The results of these *in vitro* and *in vivo* studies indicate that the protective effect of anthocyanin on the heart comes from two aspects: its indirect effect of lowering blood glucose and its direct effect on cardiac fibroblast inflammation and collagen production. The main mechanism is related to the inhibition of miR-214-mediated IL-17 expression in cardiac fibroblasts induced by high glucose. We believe that anthocyanin could be used as a preventive measure to protect the cardiac function that is impaired in diabetes. Nevertheless, the underlying mechanism of its action would be interesting to explore and would provide some significant insights. Results from echocardiography as well as HE and Masson staining showed that after administering anthocyanin, the percentage of LVEF and LVFS was restored and collagen deposition was improved compared to that in the DM group, which suggested that anthocyanin could improve the impaired cardiac function in diabetic mice. Furthermore, anthocyanin exhibited anti-fibrotic ability due to its role in attenuating inflammation and fibrosis in DM mice. However, inflammation is one of the reasons for myocardial fibrosis. Therefore, it still needs to be investigated whether the anti-fibrotic effect of anthocyanin is because of its anti-inflammatory effect.

Based on the above evidence, we further explored the underlying mechanism of action of anthocyanin in HG-induced cardiac fibroblasts. IL-17 is a pro-inflammatory cytokine produced by Th17 cells, among others. It is recognized for its role in numerous chronic inflammatory disorders (Beringer et al., 2016), including rheumatoid arthritis (Kim et al., 2017), allergic rhinitis (Piao et al., 2020), and others (Ueyama et al., 2017; Bunte and Beikler, 2019). Meanwhile, accumulating studies have demonstrated the important role of IL-17 in fibrosis. Valente et al. showed that IL-17 stimulates cardiac fibroblast proliferation and migration via the negative regulation of dual-specificity phosphatase MKP-1/DUSP-1 (Valente et al., 2012). In addition, Zhang et al. proved that ablation of IL-17 alleviated cardiac interstitial fibrosis and improved cardiac function via inhibiting long non-coding RNA-AK081284 (Zhang et al., 2018). Therefore, we wanted to explore the role of IL-17 in DM-related fibrosis in this study. IL-17 usually works synergistically with other inflammatory cytokines, such as TNF-α, IL-1β, and interferon-gamma (IFN-γ), to promote the production of inflammatory mediators, including IL-6 and IL-8 (Afzali et al., 2007; Onishi and Gaffen, 2010). Therefore, to assess the inflammatory process, we detected the expression levels of IL-17, IL-1β, TNF-α, and IL-6 in this study. We found that the expression of these four factors elevated in HG-induced cardiac fibroblasts and decreased after administration of anthocyanin. Our previous studies have confirmed the cardioprotective effect of anthocyanin, which could attenuate myocardial ischemia in mice and improve cardiac function through the ROS-JNK-Bcl2 pathway (Syeda et al., 2019). Oxidative stress also plays an important role in DCM and is associated with the apoptotic process. Oxidative stress is characterized by the excessive generation of ROS. Nicotinamide adenine dinucleotide phosphate (NADPH) oxidase is a major source of ROS (Li et al., 2019a). Superoxide dismutase (SOD) is one of the major antioxidants acting against ROS. Loss of SOD activity increased ROS production and exaggerated oxidative damage (Liu et al., 2019a). Therefore, we tested the expression of NOX4, a major NADPH oxidase isoform, and SOD2 both *in vivo* and *in vitro*. The results showed that anthocyanin could decrease the expression of cleaved caspase-3 and NOX4 and increase the expression of SOD2 compared to that in the NG or DM group (Supplementary Figure S3), indicating that anthocyanin could alleviate oxidative stress and apoptosis.

miRNAs are short non-coding RNAs that participate in the regulation of the inflammation process (Ando et al., 2013; Fu et al., 2020). It has been proven that some miRNAs in association with IL-17 regulate the inflammation process; miR-146a inhibits IL-17-mediated skin inflammation (Srivastava et al., 2017), while miR-409-3p and miR-1896 co-operatively participate in IL-17-induced inflammatory cytokine production (Liu et al., 2019b). Based on the above evidence and findings of our previous study (Yang et al., 2018), we hypothesized that the inhibitory effect of anthocyanin on IL-17 might be mediated by miR-214-3p. Therefore, we carried out AMO-214-3p transfection in cardiac fibroblasts pretreated with anthocyanin under HG conditions and found that the positive effect of anthocyanin was reversed. These results demonstrated that anthocyanin played an anti-inflammatory and anti-fibrotic role *in vitro*.

**FIGURE 6:**
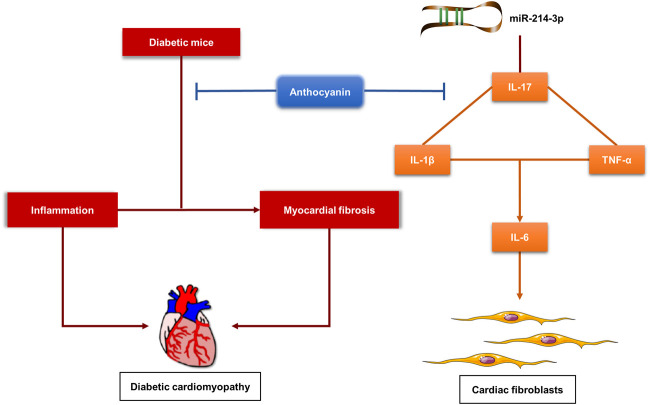
Proposed model for the function of anthocyanin in alleviating cardiac inflammation and fibrosis in diabetic mice, and regulating inflammation and fibrosis via interaction between miR-214-3p and IL-17 in high-glucose-treated cardiac fibroblasts. HG, high glucose (25 mM), CFs, cardiac fibroblasts.

In conclusion, we first found that anthocyanin could improve cardiac function by alleviating cardiac inflammation and fibrosis in diabetic mice. Then, we demonstrated that anthocyanin exhibits its anti-inflammatory and anti-fibrotic abilities through the interaction between miR-214-3p and IL-17 in HG-induced cardiac fibroblasts (Figure 6). This indicates that anthocyanin has preventive and therapeutic potential in the treatment of DM-related complications, such as DCM.

## Data Availability Statement

The original contributions presented in the study are included in the article/Supplementary Material, further inquiries can be directed to the corresponding authors.

## Ethics Statement

The animal study was reviewed and approved by the Ethics Committee of Harbin Medical University (IRB3006619).

## Author Contributions

YB and EY conceived the study; EY designed the research; EY, YY and XW conducted experiments; BY and YB supervised the experiments; EY and YY performed data analysis; EY, YY and BL wrote or contributed to the writing of the manuscript.

## Funding

This project was supported by the National Natural Science Foundation of China (Grant No. 81673426 to YB, 81730026 to BY and 81803012 to BL); Higher Education Reform Research Project of Heilongjiang Province (Grant No. SJGY20180318 to YB); and Natural Science Foundation of Heilongjiang Province (LH2019H003 to YB).

## Conflict of Interest

The authors declare that the research was conducted in the absence of any commercial or financial relationships that could be construed as a potential conflict of interest.
