# Supplementary figures and images for: Anthocyanin Protects Cardiac Function and Cardiac Fibroblasts From High-Glucose Induced Inflammation and Myocardial Fibrosis by Inhibiting IL-17
Source: Front Pharmacol. 2021 Feb 2;11:593633. doi: 10.3389/fphar.2020.593633 (PMC7884999; doi:10.3389/fphar.2020.593633)

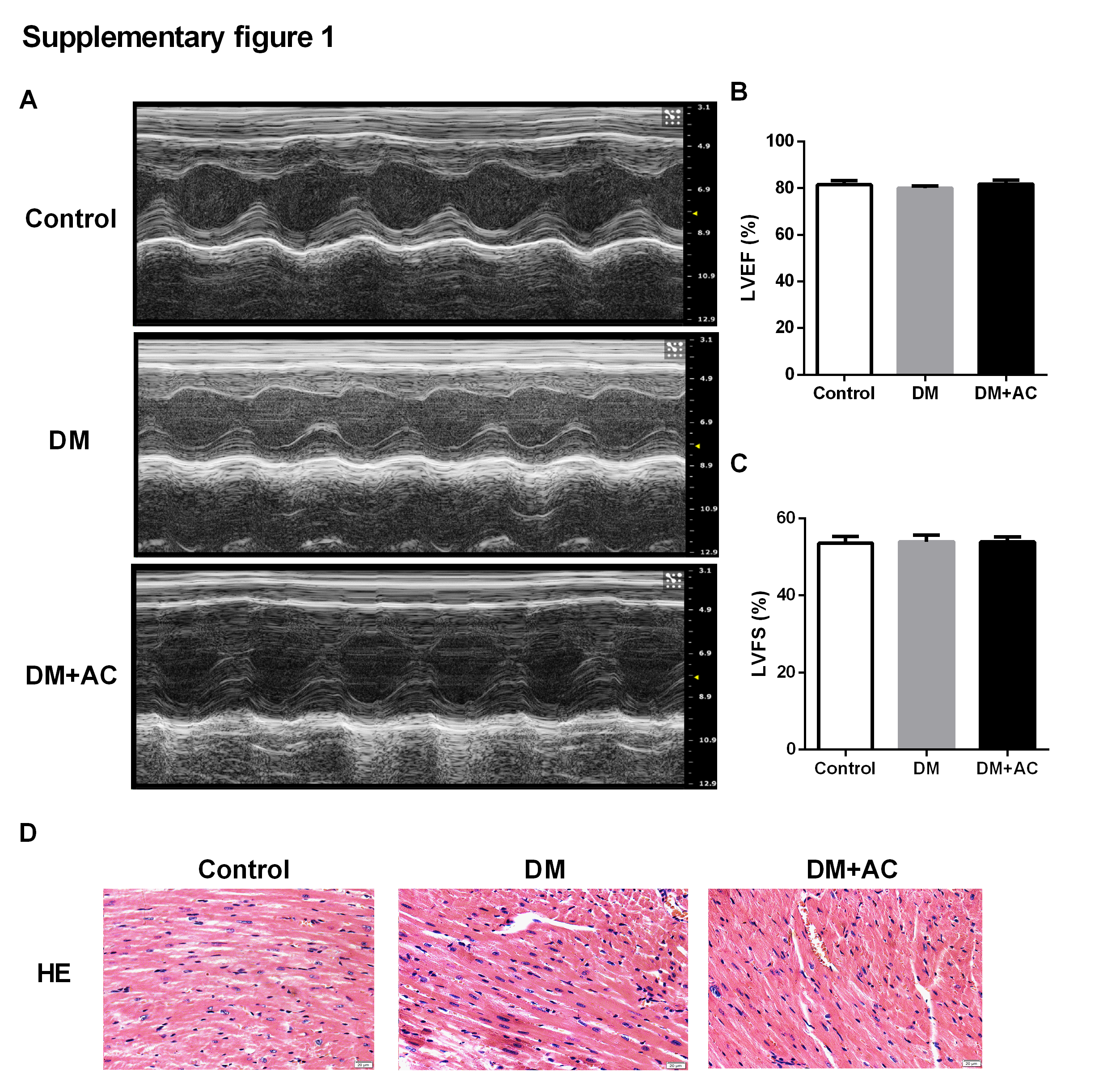

Supplement: Supplementary file 1 [file image1.tif]
